# Asymmetric Cortical Adaptation Effects during Alternating Auditory Stimulation

**DOI:** 10.1371/journal.pone.0034367

**Published:** 2012-03-28

**Authors:** Alfredo Brancucci, Giulia Prete, Elisa Meraglia, Alberto di Domenico, Victor Lugli, Barbara Penolazzi, Luca Tommasi

**Affiliations:** 1 Department of Biomedical Sciences, ‘G. d'Annunzio’ University of Chieti and Pescara, Chieti-Pescara, Italy; 2 Department of Neuroscience and Imaging, ‘G. d'Annunzio’ University of Chieti and Pescara, Chieti-Pescara, Italy; 3 Department of Psychology, ‘Alma Mater Studiorum’ University, Bologna, Italy; Hangzhou Normal University, China

## Abstract

The present study investigates hemispheric asymmetries in the neural adaptation processes occurring during alternating auditory stimulation. Stimuli were two monaural pure tones having a frequency of 400 or 800 Hz and a duration of 500 ms. Electroencephalogram (EEG) was recorded from 14 volunteers during the presentation of the following stimulus sequences, lasting 12 s each: 1) evoked potentials (EP condition, control), 2) alternation of frequency and ear (FE condition), 3) alternation of frequency (F condition), and 4) alternation of ear (E condition). Main results showed that in the central area of the left hemisphere (around C3 site) the N100 response underwent adaptation in all patterns of alternation, whereas in the same area of the right hemisphere the tones presented at the right ear in the FE produced no adaptation. Moreover, the responses to right-ear stimuli showed a difference between hemispheres in the E condition, which produced less adaptation in the left hemisphere. These effects are discussed in terms of lateral symmetry as a product of hemispheric, pathway and ear asymmetries.

## Introduction

The neural processing of sensory information is strictly related to stimulation context. When a stimulus is presented repeatedly, brain responses usually decrease in amplitude, an effect which has been characterized as neuronal adaptation [1], and which is considered to be at the basis of perceptual habituation [2]. Both processes can be considered as primitive forms of learning [3]. Auditory short-term adaptation concerns in particular the decrease, lasting few seconds, of the most reliable component of the auditory evoked potentials, namely the N100, which is assumed to reflect a complex generation process at neural level [4]–[7]. Conversely, long-term adaptation involves in general the amplitude decrease over longer periods of time, from minutes to hours, in accordance with the observation that stimulus exposure alters the way sound is encoded in the human brain [8]. The amplitude of the N100 is strongly influenced by physical features of the acoustic stimulus as the onset [9], and by psychological features such as experience (e.g. musical expertise [10], [11]). Its main generator, located in the auditory cortex, is associated with cognitive processes such as memory [12] or stimulus classification [13]. The name ‘N100’ reflects the negative polarity of the response at the vertex and its latency of about 100 ms from stimulus onset. The N100 is succeeded by the P200, whose function and meaning are less well understood [14].

Short-term adaptation has been investigated both by means of electroencephalography (EEG) and magnetoencephalography (MEG). The adaptation effects found with EEG and MEG recordings are strikingly similar to those shown by firing of single neurons observed in primary auditory cortex in response to repetition of sounds, called stimulus-specific adaptation [15]. Both occur without overt attention to sounds, are stimulus specific, and develop rapidly. In addition, repetition effects have also been observed in stimulus-induced oscillatory activity at gamma and beta frequencies [16]. Remarkably, response decrement as the main characteristic of adaptation constitutes also a methodological problem in studies on evoked responses based on the averaging procedure which need intrinsically a repetition of the stimulation to achieve reliable data.

It has been shown that when stimulus repetition occurs with another special feature of stimulation context, i.e. with the alternation of one of the parameters of sound, the adaptation effects show particular properties. Butler [17] compared the EEG responses to sounds delivered from a single loudspeaker with those evoked by sounds delivered alternately from two loudspeakers with 90° separation in the horizontal and vertical plane. When compared with response amplitudes to sounds originating always at the same location, a significant amplitude increment generated by alternating sounds was observed. In a more recent study, Yagcioglu and Ungan [18] presented alternating tones of different frequencies and found an attenuation of the response in comparison to sounds presented without alternation. However, despite alternation plays several roles in perception and cognition [19]–[26], to our knowledge research reports specifically exploring neurophysiological bases of alternating auditory stimulation are limited to the two ones mentioned above, and the issue of a possible differential role of the two hemispheres in the processing of alternating stimuli has not been faced until now. Exceptions are the studies investigating auditory stream segregation which have purposes differing from the present ones [27].

This study aims at disentangling the role of some parameters of alternating acoustic stimulation which could play a role in the adaptation process, such as ear of presentation and tone frequency. In particular, the interest is focussed on the possible different adaptation processes during alternation that might occur for these parameters in the two hemispheres. Do left and right auditory cortices adapt in the same way when stimuli are presented at the contralateral or ipsilateral ear? Does frequency alternation of the presented tones play a role in this process, either by itself or by interacting with the ear of presentation? The working hypothesis is formulated according to the current view of hemispheric specialization, which is parameter-specific structured [28] and points to a physical dichotomy which assigns a better spectral resolution to the right auditory cortex and a better temporal resolution to the left auditory cortex (cfr. the asymmetric sampling theory [29]–[32]). Due to the different physical features of the stimuli, it is expected that the right hemisphere would exhibit different adaptation effects compared to the left in particular when tone frequency comes into play. Due to the localization of primary and secondary auditory cortex, central areas are expected to be more involved than anterior or posterior areas in such processes.

## Materials and Methods

### Ethics statement

All subjects gave their written informed consent according to the Declaration of Helsinki (World Medical Association, 1991) and could freely request an interruption of the investigation at any time. The local Institutional Ethics Committee (University of Chieti and Pescara, Italy) approved the general procedures.

### Subjects

Fourteen young (mean age ± standard error = 23±0.56 years) healthy volunteers (university students, 8 males and 6 females) participated in the present study. None of them was musician and none declared to have auditory impairment. In addition, audiometric assessment was performed, in which subjects had to press a button when a complex tone of 264 or 395 Hz, presented via earphones repeatedly with increased intensities (steps of 2.5 dBA), became perceivable. Subjects were recruited when no (±5 dBA) different hearing thresholds were present between left and right ear. Average handedness was 71.6±14.4 (Edinburgh Inventory corrected according to Peters [33]) with one subject scoring <0 (−40) and six subjects scoring 100.

### Stimuli

Acoustic stimuli were delivered by means of headphones (Philips e SHP5400). They were composed of two monaural pure tones having a frequency of 400 and 800 Hz, duration of 500 ms and intensity of 65 dBA. Tones were arranged in sequences of 12 s duration with no interstimulus interval (24 stimuli per sequence), composed as follows:

in the first condition stimuli were presented as in a classical evoked potential session. Left and right stimuli of 800 and 400 Hz frequency were presented in a random order within each block (EP condition).
in the second condition (alternation of frequency and ear, FE condition) stimuli alternated between both ears and frequencies, for example: 800 Hz right ear −400 Hz left ear −800 Hz right ear −400 Hz left ear, and so on.
in the third condition (alternation between frequencies, F condition) stimuli alternated only between frequencies, and were presented only to one ear within one sequence. For example: 800 Hz left ear −400 Hz left ear −800 Hz left ear −400 Hz left ear, and so on.
in the fourth condition (alternation between ears, E condition) stimuli alternated only between ears, whereas frequency remained constant. For example: 800 Hz right ear −800 Hz left ear −800 Hz right ear −800 Hz left ear, and so on.

All parameters (400 and 800 Hz tones, ear of presentation) were completely counterbalanced both between and within conditions i.e., for instance, in the E condition sequences were composed half the times of 400 Hz tones and half the times of 800 Hz tones and started half the times with the left and half the times with the right ear. Thirty-two sequences were presented for each condition and the whole experiment lasted about 35 minutes.

### EEG recordings

Subjects were sitting on a comfortable chair, wearing a pair of headphones. The headphones were worn in inverted position by half of the subjects in order to keep possible acoustic differences in the ear pieces controlled. No task was requested, except to maintain a stable level of attention throughout the entire recording period. A brief training session served to minimize blinking and eye movements. AEPs were recorded from 32 EEG electrodes placed according to the 10–20 International system for EEG electrode positioning, using a Professional BrainAmp MR EEG amplifier. Electrode sites were the followings: Fz, Fp1, Fp2, F3, F4, F7, F8, Fc1, Fc2, Fcz, Fc5, Fc6, T7, T8, CP5, CP6, Pz, P3, P4, P7, P8, Cz, C3, C4, Cp1, Cp2, Oz, O1, O2, PO3, PO4, POz.

The electrode impedance was kept lower than 10 KOhm. To monitor blinking and eye movements we recorded electrooculography (EOG) from the left eye [34]. EEG recording parameters for all data were filtered with a 0.05–100 Hz bandpass filter, sampling rate of 1000 Hz, and acquisition time from −50 ms to +500 ms with respect to the onset of the auditory stimulation. Averaged AEPs resulted from 768 auditory stimuli for each condition.

### EEG data analysis

EEG single trials contaminated by blinking, eye movements, and involuntary motor acts were rejected offline (reference threshold: ±75 µV). The spatial resolution of artefact-free EEG data was enhanced by surface Laplacian estimation (regularized 3-D spline function), which reduces low spatial frequencies of EEG distribution possibly due to head volume conductor effects. This data analysis method uses information from all 32 electrodes in order to spatially enhance the potentials at the sites of interest. It annuls electrode reference influence and requires no arbitrary computational assumptions [35]. Surface Laplacian estimation has been successfully used in previous studies with sensory paradigms [36]–[40] and can be considered a valid tool together with others, such as equivalent dipole localization or linear inverse estimation. However, ERP sources must be inferred with caution since surface Laplacian maxima could not fit the corresponding tangential cortical sources. Here, we accounted for such a limitation by considering wide scalp frontal, central, and posterior areas of interest both in the left and right hemisphere.

The baseline for the measurements of the AEP was taken during the period of 50 ms preceding stimulus onset and data were analyzed at the latencies of the observed peaks (N100 and P200). Amplitude was taken as the peak response for each component. The peak response is intended as the exact voltage value of the sample corresponding to the maximum of the evoked response, for each component.

To obtain EEG data from the regions of interest, the scalp was subdivided in 6 regions of interest (left and right hemisphere) using the following electrode distribution:

Anterior left: FC5, F3

Anterior right: FC6, F4

Central left: Cp5, C3

Central right: Cp6, C4

Posterior left: P3

Posterior right: P4

**Figure 1:**
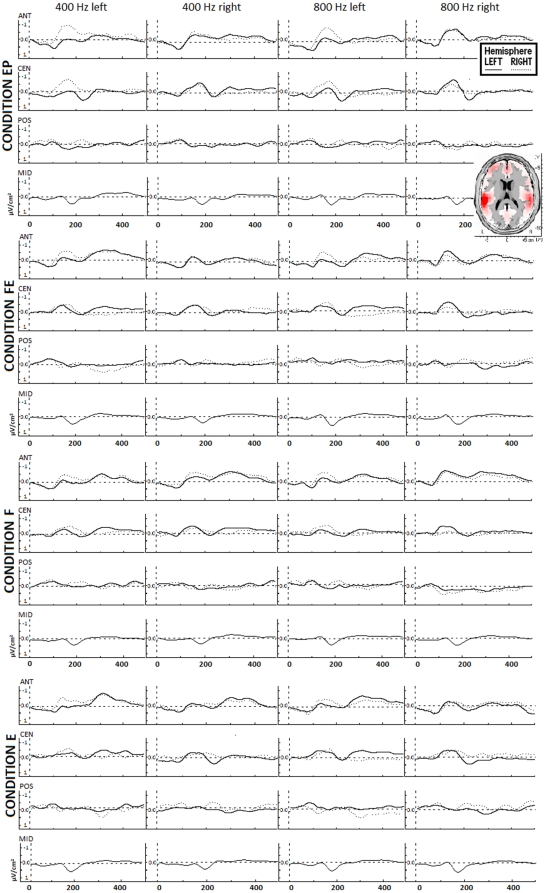
Mean laplacian EEG responses from anterior, central, and posterior areas to the 4 acoustic stimuli in the 4 conditions (all subjects). Each graph shows response from the left and right hemisphere. EP = control condition (evoked potentials); FE = alternation of frequency and ear; F = alternation of frequency; E = alternation of ear. *Top right*: LORETA map showing the main neural sources in the interval 100–350 ms (all stimuli, all conditions).

Of note, surface laplacian estimation uses anyway information from all 32 electrodes to spatially enhance the potentials at the electrodes of interest. On average, the mean (± standard error) number of individual artefact free data was of 178.6±3.2 single trials for the EP condition, 173.0±6.0 single trials for the FE condition, 177.0±3.4 single trials for the E condition, and 179.7±3.9 single trials for the F condition (differences between conditions were not significant according to analysis of variance). Mean EEG traces for each stimulus in each condition are depicted in Fig. 1, which represents also the main neural sources (LORETA map) of the recorded signals, located in BA 41–42. LORETA map has been computed on grand-mean data from all conditions (all stimuli) in the time interval from 100 to 350 ms post-stimulus. For sake of completeness, the data from Fz, Cz, Pz, and Oz were also evaluated, in a separate analysis.

### Statistical design

Dependent variables were amplitude and latency of EEG responses. On both variables, two 4×2×2×2 analyses of variance (ANOVAs) were performed, one for the first and one for the second AEP component, with the following factors: Condition (EP, FE, E, F), Tone frequency (400 Hz, 800 Hz), Ear (left, right), and Hemisphere (left, right). Post-hoc comparisons were calculated using Duncan test.

## Results

### Amplitude

At N100 latency, anterior areas showed an interaction Ear×Hemisphere (F = 7.32; p = 0.02; partial eta squared = 0.38) due to a stronger response in the right hemisphere to tones presented at the left ear (p = 0.01). Conversely, in the left hemisphere no differences related to the ear of presentation were observed. A further significant interaction was Condition×Ear (F = 3.29; p = 0.03; partial eta squared = 0.21) due to stronger responses to stimuli presented at the left ear in the EP, compared to the other conditions (FE, F, E: p<0.01) and within the EP condition in comparison with stimuli presented at the right ear (p<0.01). For stimuli presented at the right ear the response to the EP condition was stronger compared to the FE condition (p = 0.01).

**Figure 2:**
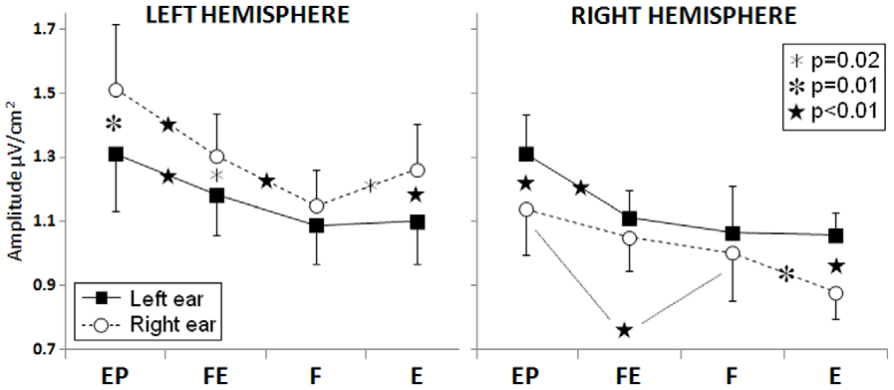
Group mean responses (N100) from central areas showing the main results of the study. Asterisks denote significant post-hoc results. X-axis labels refer to the experimental conditions: EP = control condition (evoked potentials); FE = alternation of frequency and ear; F = alternation of frequency; E = alternation of ear).

Central areas showed a main effect of Condition (F = 6.67; p<0.01; partial eta squared = 0.35) due to stronger responses in the EP compared to the F, E (p<0.01), and FE (p = 0.02) conditions. Interaction effects were found between Ear and Hemisphere (F = 13.2; p<0.01; partial eta squared = 0.52), due to stronger responses to the contralateral ear in each hemisphere (left hemisphere: p = 0.02, right hemisphere: p = 0.04), between Tone frequency and Ear (F = 11.6; p<0.01; partial eta squared = 0.49) due to stronger responses to the 400 Hz tone presented at the right compared to left ear (p = 0.02) and to stronger responses to 800 Hz compared to 400 Hz tones presented at the left ear (p = 0.01), and between Condition, Ear and Hemisphere (F = 3.67; p = 0.02; partial eta squared = 0.23, see Fig. 2 for post-hoc results).

Posterior areas showed an interaction Tone frequency×Hemisphere (F = 7.88; p = 0.01; partial eta squared = 0.39) due to a stronger response in the right hemisphere to 800 Hz compared to 400 Hz tones (p = 0.01), and to a stronger response to the 800 Hz tone in the right compared to the left hemisphere (p<0.01).

Concerning midline electrodes, a Tone×Condition interaction was observed (F = 3.29; p = 0.03; partial eta squared = 0.21) due to a stronger response to the 400 Hz tone in the EP compared to all other conditions (p<0.01), to a stronger response to the low compared to high tone in the FE condition (p<0.01), and to a stronger response to the high tone in the FE compared to F condition (p = 0.01). A further significant interaction was Tone×Ear (F = 12.67; p<0.01; partial eta squared = 0.51) due to a weaker response to the 400 Hz tone presented at the left compared to right ear (p<0.01) and compared to the 800 Hz tone presented at the left ear (p<0.01).

At P200 latency, anterior areas showed an interaction Ear×Hemisphere (F = 5.76; p = 0.03; partial eta squared = 0.32) due to stronger responses produced by left compared to right ear tones in the right hemisphere (p<0.01).

### Latency

Mean latencies of the first component (N100) were 129±3 ms for anterior areas, 110±2 ms for central areas, and 126±3 ms for posterior areas. Central areas showed an interaction Tone frequency×Ear (F = 6.04; p = 0.03; partial eta squared = 0.33) due to shorter latency of the response to the 400 Hz tone presented at the right compared to the left ear (p = 0.02) and to a shorter latency of the 400 Hz compared to the 800 Hz tone presented at the right ear (p = 0.02).

Mean latencies of the P200 were 306±4 ms for anterior areas, 298±4 ms for central areas, and 318±4 ms for posterior areas. Anterior areas showed an interaction Condition×Ear (F = 3.74; p = 0.02; partial eta squared = 0.24) due to slower responses to the stimuli presented at the left ear in the EP compared to the FE (p<0.01) and E conditions (p = 0.04). Central areas showed a main effect of Condition (F = 4.77; p<0.01; partial eta squared = 0.28) due to an adaptation (delay) of the response in the FE and F (p<0.01) as well as E (p = 0.03) condition compared to the EP condition.

Posterior areas showed an interaction Condition×Ear (F = 2.98; p = 0.04; partial eta squared = 0.20) due to earlier response to the FE compared to the EP condition with tones presented at the right ear (p = 0.02). Finally, midline areas showed an interaction Condition×Ear (F = 3.28; p = 0.03; partial eta squared = 0.21) due to faster responses to tones presented at the right ear in FE compared to the other conditions (p = 0.02).

## Discussion

The results of the present study can be divided into three points: *a)* adaptation effects can be different in the two hemispheres, *b)* during passive listening, functional asymmetries in the auditory domain have to be ascribed also to the stimulated ear (or auditory pathway) other than to the hemisphere, and *c)* the canonic “contralaterality” of the auditory system can be altered by stimulus alternation.

Regarding to the first (main) point, in accordance with previous evidence [17], [18] the present study showed that alternating stimulation produces adaptation with specific spatiotemporal characteristics in the brain. At N100 latency, the areas which underwent adaptation were located mainly in the central cortex (roughly, temporal and lateral parietal lobes) where, other than a general reduction of the response which occurred with all three types of alternation presented, also an asymmetrical pattern of adaptation was observed. In the left hemisphere, responses underwent to adaptation in all patterns of alternation for monaural tones presented at the left and at the right ear. Conversely, in the right hemisphere the tones presented at the right ear during simultaneous alternation of frequency and ear produced no adaptation. Moreover, the responses to right-ear stimuli showed an opposite pattern in the two hemispheres during simple alternation of ear, in that less adaptation in the left central areas and more adaptation in the right central areas were observed, compared to simple alternation of frequency. Of note, central areas showed adaptation with all alternating stimulation patterns also at the second component (“P200”), in terms of a delay of the response. N100 adaptation due to alternation was also detected in the anterior (frontal) cortex for stimuli presented at the left ear, but it was negligible for stimuli presented at the right ear. In general the adaptation was weaker when the stimulus changed both between ears and in frequency. Thus, the present study showed that hemispheric functional asymmetries can be detected also from the viewpoint of neural adaptation processes.

In the framework of adaptation processes investigated in the two hemispheres, an issue emerges which is related to the meaning of such processes: a neural structure which adapts is enhancing its analysis of the stimulus or the analysis becomes more superficial? It should be considered that adaptation might be a neural correlate of priming, which refers to improved processing of a repeated stimulus according to some behavioral measure, e.g. greater accuracy in identifying the stimulus, or faster response times to make a decision about it. It is important to note that, under certain conditions, priming can be associated with increased activity, rather than reduction [41], [42]. However, concerning hemispheric specialization, on the basis of the current view, if adaptation reflects an enhanced analysis of the stimulus, one would expect stronger adaptation to frequency in the right hemisphere. This was not the case here, as the right hemisphere showed less adaptation than the left, albeit only for the stimuli presented in the right ear. In a previous EEG study in which visual stimuli were presented [43], responses to faces showed more adaptation in the right hemisphere whereas responses to words showed no asymmetric adaptation processes. Future studies specifically aimed at disentangling this point could provide more precise answers to this issue.

Regarding to the second point, the present results showed that during monaural stimulation the functional asymmetries in the auditory domain at N100 latency depend upon an interaction among the ear, the auditory pathways and the hemispheres, and not only from the hemispheres. Fig. 2 shows that the monaural input from the left ear does not give rise to activation differences in the two hemispheres in terms of amplitude, whereas the same input from the right ear gives rise to stronger responses in the left hemisphere, in all conditions. Moreover, while the signals from both ears undergo adaptation in both hemispheres in all conditions with respect to the control condition, comparing the three alternation conditions it can be observed that whilst the input from the left ear does not show any differences, the input from the right ear undergoes stronger adaptation in the right hemisphere in the E condition and in the F condition in the left hemisphere. Although it is not possible to univocally disentangle the contribution to the EEG signal arising from the different stages of auditory processing with the present technique, the pattern of results observed here strongly point to the existence of a left-right asymmetry which is not confined to the cerebral cortex. This result is in accordance with several previous findings on the auditory system [44]–[47]. In a magnetoencephalography study on auditory cortical responses to dichotic speech stimuli, Della Penna and coworkers [48] showed that the notion of auditory cortical asymmetries has to be integrated at least to the ascending auditory pathways. They showed that, concerning the left hemisphere, specialized for speech, during dichotic listening of syllables the ipsilateral pathway is strongly inhibited, thus favoring the perception of the input to the right ear. Conversely, concerning the right hemisphere both pathways are inhibited to the same extent. In this framework (speech stimuli), the privileged ear is the right one, the input of which can reach the left hemisphere via a preferential route that suppresses the ipsilateral left auditory pathway. In concomitance, the input to the right ear can reach the right auditory cortex without significant loss of information compared with the input of the left ear, and from there it can be sent to the speech areas of the left auditory cortex via corpus callosum.

The third point implicates the notion of lateral symmetry i.e., in the case of the auditory system, the fact that usually the major response in each hemisphere is observed to sounds presented contralaterally rather than ipsilaterally. This fact is known since long time in auditory neuroscience. About 30 years ago, Elberling [49] and Reite [50] with coworkers found the magnetic responses obtained with contralateral stimulation to occur earlier and with a greater amplitude than those obtained with ipsilateral stimulation. A subsequent study [51] further showed that at 100 ms latency the magnetic, but not the electrical response to contralateral stimuli is approximately 38% stronger and 10 ms earlier than the response to ipsilateral stimuli. In the present study, condition-independent auditory lateral symmetry was observed at N100 latency in central areas of both hemispheres and at P200 latency in right anterior areas also with electrical recordings although only in terms of amplitude, as shown by the results of the control condition. Furthermore, stimulus manipulation in terms of alternation showed that such differences between contralateral and ipsilateral responses to auditory stimuli can be altered by stimulation context (condition-dependent lateral symmetry). Indeed, whereas lateral symmetry could be observed in the control and in the ear alternation condition, when only frequency alternated it was no longer observable, and when both ear and frequency alternated, it was observable only in the left hemisphere.

Finally, minor results concerning differential responses to tone frequencies deserve a short discussion. At central areas, the 400 Hz tone produced a stronger and earlier N100 when presented at the right compared to left ear; of the stimuli presented at the left ear the 800 Hz tone produced a stronger response than the 400 Hz tone; and of the stimuli presented at the right ear the 400 Hz tone produced a faster response compared to the 800 Hz tone. At posterior areas the right hemisphere produced stronger responses to 800 Hz compared to 400 Hz tones, and the right hemisphere responses to the 800 Hz tone were stronger than those of the left hemisphere. In sum, in accordance with what postulated concerning the importance of the ear and auditory pathways, it seems that the presence of an association within tone frequency (in rough terms of high-low) and ear cannot be excluded, in the direction of a preference of the left ear for high and of the right ear for low tones. Conversely, at the cortical level this association seems to be reversed, with a preference of the right hemisphere for high tones. The relationship between tone frequency and ear of input requires further investigation, as to our knowledge no studies have addressed this issue.
